# Dengue: A review of laboratory diagnostics in the vaccine age

**DOI:** 10.1099/jmm.0.001833

**Published:** 2024-05-09

**Authors:** Jaimie L. Frazer, Robert Norton

**Affiliations:** 1 Pathology Queensland, Townsville QLD, Australia; 2 Faculty of Medicine, University of Queensland, Brisbane, QLD, Australia

**Keywords:** Dengue, laboratory diagnosis, review

## Abstract

**Background.** Dengue is an important arboviral infection of considerable public health significance. It occurs in a wide global belt within a variety of tropical regions. The timely laboratory diagnosis of Dengue infection is critical to inform both clinical management and an appropriate public health response. Vaccination against Dengue virus is being introduced in some areas.

**Discussion.** Appropriate diagnostic strategies will vary between laboratories depending on the available resources and skills. Diagnostic methods available include viral culture, the serological detection of Dengue-specific antibodies in using enzyme immunoassays (EIAs), microsphere immunoassays, haemagglutination inhibition or in lateral flow point of care tests. The results of antibody tests may be influenced by prior vaccination and exposure to other flaviviruses. The detection of non-structural protein 1 in serum (NS1) has improved the early diagnosis of Dengue and is available in point-of-care assays in addition to EIAs. Direct detection of viral RNA from blood by PCR is more sensitive than NS1 antigen detection but requires molecular skills and resources. An increasing variety of isothermal nucleic acid detection methods are in development. Timing of specimen collection and choice of test is critical to optimize diagnostic accuracy. Metagenomics and the direct detection by sequencing of viral RNA from blood offers the ability to rapidly type isolates for epidemiologic purposes.

**Conclusion.** The impact of vaccination on immune response must be recognized as it will impact test interpretation and diagnostic algorithms.

## Introduction

Dengue virus (DENV) is a positive-sense RNA virus in the genus *Flavivirus* [1]. It is transmitted by mosquitoes of genus *Aedes*, principally *Ae. aegypti* and *Ae. albopictus* [2]. It has four serotypes (DENV1-4) that have different antigenicity and phylogeny. It is widespread in tropical and subtropical regions of Asia, Oceania, the Americas and Africa [3]. DENV serotypes co-circulate in some regions, leading to reinfections and hyperendemic spread [4]. Cases and outbreaks occur in non-endemic areas where competent vectors are present [5,6]. These are occurring in increasingly higher latitudes, which likely reflects the impact of climate change and reflects the potential for future endemic range expansion [7]. DENV infection is an infection of increasing global significance.

In endemic areas, diagnostic testing to confirm that DENV is the cause of a febrile illness, and the identity of the infecting serotype is important for clinical management, epidemiology and public health reasons. The four serotypes of DENV cause a similar illness, with similar presentation to other arboviral infections and acute febrile illnesses [8,9]. The performance of clinical diagnostic criteria for DENV diagnosis have poor specificity due to similarity with other infections, therefore laboratory diagnosis is important to confirm the causative condition [10]. Severity of infection can range from asymptomatic to severe [11]. Detection of asymptomatic (reservoir) infection is important for considering vector control and other public health interventions [12]. Though primary infection with complete absence of symptoms is probably uncommon, it may be more prevalent in secondary and subsequent infection [13]. Identification of new serotypes and genotypes in a region may cause outbreaks. As immunity is serotype specific, reinfection with a different serotype increases the risk of potentially fatal severe Dengue infection through antibody-dependent enhancement (ADE) [14]. Finally, pre-transfusion screening of blood supplies can be considered in high-prevalence regions to reduce the risk of iatrogenic transmission [15]. In non-endemic areas, DENV is the most common cause of viral illness in returning travellers [16]. Therefore, diagnostic testing is an important component of DENV management.

Control and mitigation measures for DENV may impact the need for laboratory diagnostics. Control measures have had some recent successes, including the deployment of *Wolbachia*-infected mosquitos (which has significantly decreased infection rates in release areas) [17,23], and the development of vaccines [24,27]. Due to the risk of subsequent severe DENV infection related to ADE [28], vaccination is only recommended for those who have confirmed prior DENV infection, unless there is high local seroprevalence in the target age group for vaccination [27,29]. This increases the importance of obtaining an accurate diagnosis for acute febrile illness in areas where DENV is present, and for laboratory surveillance of seroprevalence, especially in areas where other control measures (such as *Wolbachia*-infected mosquito release) may change population exposure. In addition, vaccines appear to have different efficacy against different serotypes [30,31], so serotype surveillance is also important for planning vaccination programmes. As vaccination and other control measures are applied, the need for and focus of DENV diagnostics may change in each region.

This review will assess current laboratory diagnostic methods for DENV and their roles, especially in the context of the changing epidemiology of DENV and availability of vaccines. While the primary focus is testing in the diagnostic pathology laboratory, it is recognized that point-of-care (POC) methods have an important role in assessment prior to vaccination in lower prevalence areas. As the majority of the disease burden of DENV infection is in settings where advanced POC diagnostics may be helpful, selected new assays targeted at field use are also considered. The important role of reference laboratories is acknowledged, for their maintenance of reference methods less used in the diagnostic laboratory.

## Virus isolation

Detection of a virus in culture is the gold standard for identification and serotyping [32], an important component of outbreak investigation [33,34]. Peak viremia as detected by RT-PCR (a more sensitive method) occurs earlier in secondary infection than primary infection (2.4 vs 3.0 days) and twice as many patients (50 % cf 25 %) have an obvious viral peak [34]. In secondary dengue infection, duration of viremia is shorter (5 days in both children and adults, compared with 7–9 days in primary infection). Earlier specimen collection will increase the chance of successful detection of virus by isolation methods, and, correlating with this, specimens with a higher viral load by RT-PCR and lower IgM and IgG antibody titre have a higher probability of successful virus isolation [35]. Because of this, virus isolation is less likely to be successful in secondary infection [35].

Various specimens can be used including serum, plasma, CSF, and tissues derived from autopsy [36,37]. Isolation from urine and saliva has however been attempted without success [38]. Specimens are sensitive to pre-analytical handling, which impacts sensitivity. A significant decrease in culture detection of virus from serum was noted with 24 h of refrigerator storage or 15 days of freezing at −30 °C, when compared to immediate processing after retrieval from liquid nitrogen storage [39]. Because of this, many studies now use molecular assays as a gold standard instead.

Described methods of viral propagation include inoculation of larval [40] or adult mosquitos [41,42] such as *Aedes* spp. (natural vectors) or *Toxorhynchites* spp. (larger and, being unable to transmit the virus, safer) [32], or cell cultures (e.g. *Aedes albopictus* C6/36 cells, Vero cells) [32,43]. Mosquito inoculation is most sensitive as not all strains grow well in mosquito cell culture, and mammalian cells are harder to infect and more prone to mutation with serial passage [32]. The yield of virus isolation in RT-PCR positive specimens improved from 62.5 % of specimens in C6/36 cell culture to 79.4 % in intrathoracic inoculation of *T. splendens* [35]. Larval cranial inoculation yields faster positive results than adult head or thorax, but still required up to 5 days for reliable detection (compared with 7 for adult mosquitos) [40]. Intracerebral inoculation of newborn mice can be used but is considered relatively insensitive [44].

Viral culture preparations can be assessed for detection of virus with direct or indirect fluorescence assay of crushed insect preparations or cell cultures, or plaque assays of Vero cells such as the plaque neutralization reduction test (PNRT) [32,45]. Indirect immunofluorescence may be used to detect and serotype the virus [46].

Virus isolation and culture is not used in most diagnostic laboratory settings as it is expensive, laborious and time consuming [47]. Additionally, molecular amplification, detection and sequencing methods can be performed directly on clinical specimens [48,49] without requiring a virus isolation step with subsequent identification and titration. Virus isolation and culture of diagnostic specimens is mainly performed in reference laboratories where generation of antigen for test, reagent and research purposes is required, for plaque neutralization reduction testing, or where very low-level viraemia requires biological amplification prior to further testing [32]. Even in this case, virus isolation is unlikely in specimens with RT-PCR CT values>25, whereas higher CT values (up to 40) would still be considered positive in many assays [32]. Culture of circulating viruses is important for reference laboratory and public health purposes, including supporting development of future diagnostic tests, vaccines and reagents. However, the role of virus isolation and associated assays in the clinical diagnostic laboratory may be filled by more practical alternatives.

### Neutralization assays

**Fig. 1.**
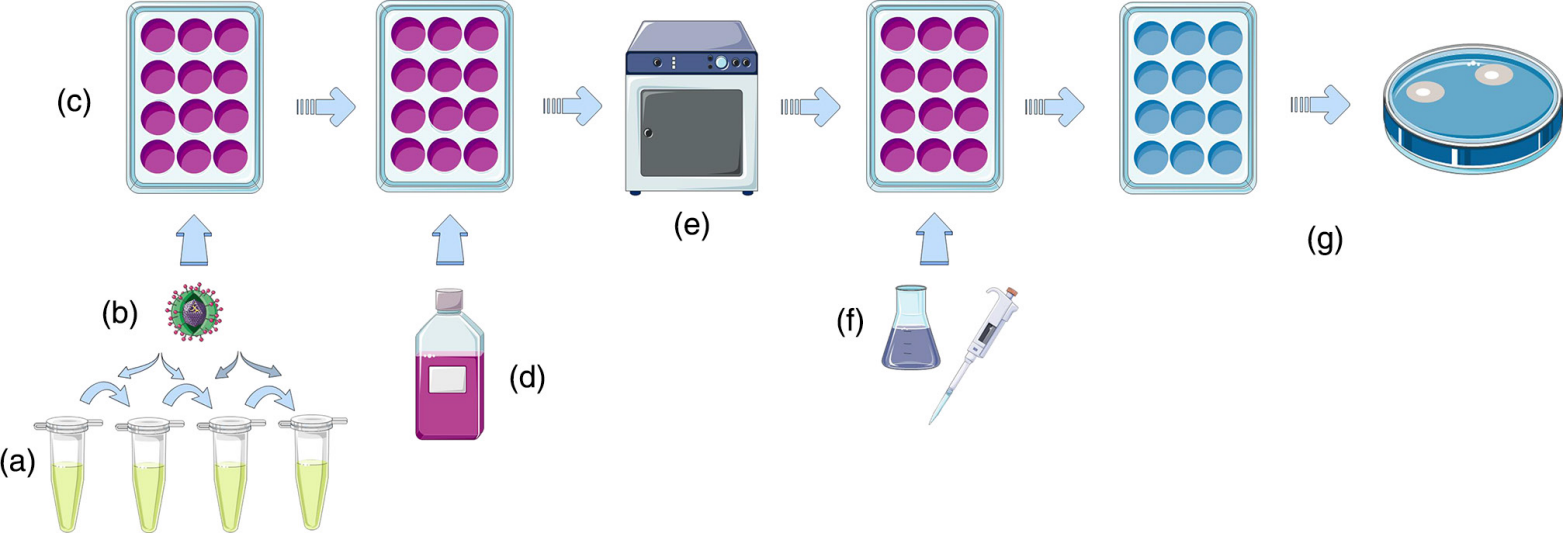
Principle of plaque neutralization reduction test. Serial dilutions are made of patient sera (**a**). A known concentration of Dengue virus is added to each diluted serum and these are incubated (**b**). The mixture is inoculated to cell cultures in a multi-well plate and incubated (**c**). A semi-solid medium is overlaid to prevent further virus dispersal (**d**). The cultures are incubated for 5–7 days (**e**). Cultures may then be stained and fixed (f) prior to assessing for the presence of plaques (g). Image created using components adapted from Servier Medical Art http://smart.servier.com used under CC BY 4.0.

PRNT in Vero or BHK cells are the original reference standard for titration of neutralizing antibody [8]. PRNT measures antibody against envelope (E) glycoprotein, which plays an important role in attachment and fusion to host cells [50]. It is technically challenging to perform, is relatively slow [51], and exhibits variability within and between laboratories [52]. A schematic is given in Fig. 1. As a result, WHO has developed guidelines to standardize the assay to particular Vero cell lines, procedures for assessment of plaque counts, and provided lists of reference viruses for each serotype, while acknowledging the need to assess wild-type viruses [50]. Some viruses form small or inapparent plaques leading to unsuitability of standard PRNT [32,53]. Focus reduction neutralization testing (FRNT) may also be used and has the advantage of higher throughput on 96-well plates and automated reading, but otherwise also has the disadvantages of PRNT [54,57].

PRNT is the reference standard used in evaluation of antibodies in immunity and vaccine response [50,52], though the presence of neutralizing antibody may not completely concur with protection from infection [58]. Neutralization tests are the most specific serologic test for primary DENV with respect to cross-neutralization with other flaviviruses [50], but for subsequent flavivirus infection or dengue reinfection, titres tend to be biassed towards the original infecting serotype [59,60]. This reduces specificity of neutralization tests in secondary DENV or other flavivirus infection [60,61].

Flow cytometry (FC) is an alternative method for enumerating cells that are positive for dengue infection and can therefore be used to evaluate neutralization [51]. This method has been used successfully on eluted dried blood spots as well as sera [57]. The originally described FC method used mosquito cell lines and monoclonal antibodies (mAb) (e.g. 4G2, which binds to dengue eE protein) to label infected cells following incubation with serial dilutions of neutralizing patient sera. This yielded comparable results to PRNT in Vero cells (though was insensitive with low titres) [51] but with the advantage of speed as it could be performed at 24 h rather than 4–7 days. Subsequent developments of FC using Vero cells and U397 human monocytes [53] or Raji B-cells [52] that express the DENV attachment factor DC-SIGN (which increases the susceptibility of cells to DENV infection 10-fold) and 2H2 (preM-Ag) as the mAb label found comparable results. These refinements expanded the approach to higher-throughput methods and had the benefit of using human cells to optimize the applicability to vaccine development. The assay showed improved reproducibility when compared with PRNT and serotype specificity was similar or better [62].

Further development of FC for neutralization includes the use of plasmid-produced pseudo-infectious viral replicas (reporter virus particles, RVPs). These remove the requirement for monoclonal antibody labelling of infected cells prior to FC as infected cells will express an intrinsic reporter. They have the additional benefits of not requiring live virus. The method performed comparably to traditional PRNT and had good inter- and intra-laboratory reproducibility [63]. Access to flow-cytometry equipment and appropriate mAbs and RVPs will limit the applicability of these assays to research settings in the near future.

## Ns1 antigen detection

Non-structural protein 1 (NS1) is a highly conserved viral glycoprotein, expressed in both membrane-associated and secreted forms, which can be used in rapid diagnostic and serologic tests [64,67]. It is detectable in early infection (generally from days 1–9 of symptoms, although it has been recorded as late as day 18 [68,69] and can exceed the duration of viremia [70,71]. In secondary DENV, NS1 is paradoxically detectable at higher concentrations [72]. This is possibly due to increased viral cell mass due to increased cellular uptake and intracellular replication from antibody-dependent enhancement. It is, however, for a shorter duration (5–7 days) than in primary dengue, possibly due to an anamnestic immune response resulting in formation of immune complexes with IgM and IgG [34,70, 73].

**Fig. 2.**
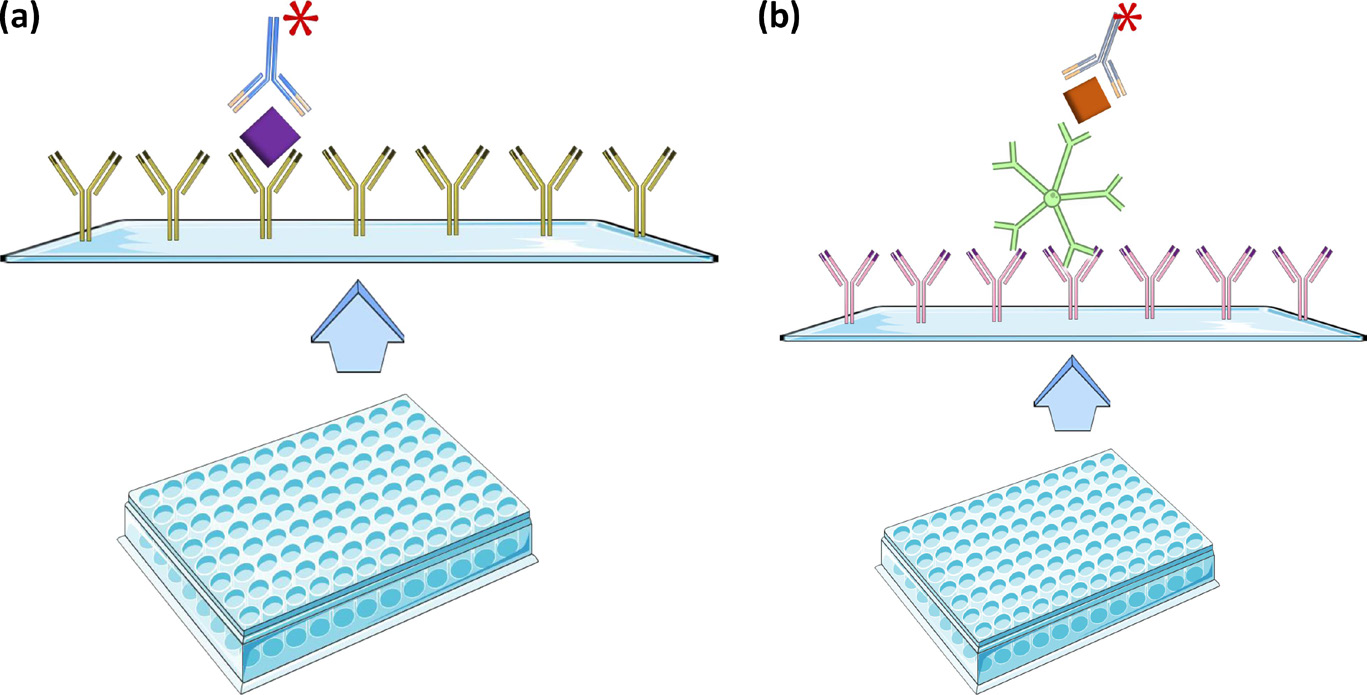
Principles of common diagnostic serology assays. (a): NS1 antigen ELISA. A plate is coated with anti-NS1antibodies (green). NS1 (purple) from the patient’s serum is captured. HRP-conjugatedNS1 antibody (blue) is added to allow detection by colour change. (b): IgM capture antibody ELISA. A plate is coated with anti-IgMantibodies (pink). This captures IgM (green) in the patient specimen.Dengue virus antigen is then added (orange), and is captured by the IgM antibody. HRP-conjugateddengue antibody (blue) is added to allow detection by colour change. Image created using components adapted from Servier Medical Art http://smart.servier.com used under CC BY 4.0.

NS1 antigen detection is available in commercial assays by antigen capture sandwich EIAs, and in immunochromatographic tests (ICTs) [66]. A schematic for the EIA is given in Fig. 2a. Sensitivity is 94 % relative to viral culture^74(p20)^. Relative to PCR, sensitivity is 87–88 % for EIA and 81 % for ICTs [66]. Sensitivity is higher in patients without IgM and IgG at time of testing [70]. This probably relates to humoral immunity reducing the quantity of soluble NS1 antigen available to react with immunoassays. Field assessments confirmed high specificity of both EIA and ICT in regions where other arboviruses and febrile illnesses such as leptospirosis and typhoid are present [70]. This concurs with a study that found potential NS1 antigen nonspecificity between flaviviruses was unlikely to be significant with currently available tests [74]. Four meta-analyses of two commercial NS1 Ag ELISAs and two commercial ICTs on clinical specimens with variable inclusion criteria and comparator specimen found lower pooled sensitivity of approximately 66 % with significant heterogeneity, but higher (77–94 %) pooled sensitivity when only primary infection is considered [75,77]. A more recent meta-analysis of 14 brands of commercially available NS1 ICT found similar pooled sensitivity for NS1, and sensitivity of 90 % if a combination of NS1, IgM and IgG ICT was used [78] Two meta-analyses reported ICTs were more sensitive than ELISA, but this was not a statistically significant difference in one study, and heterogenous comparators were used [75,76]. Specificity was greater than 97 % [75,79].

While some studies have found different sensitivities of detection of NS1 antigen by infecting serotype, others have not [64,66, 70, 75, 77, 79,84]. The serotypes performing better or worse than others vary between studies, though DENV2 and DENV4 were commonly mentioned. This may relate to differences in prior DENV exposure in the populations concerned. This is because NS1 antigen appears to be less sensitive in secondary DENV. This could relate to a shorter window of detection, true lack of specificity due to polymorphism of NS1 or reduced circulating levels of NS1 in some serotypes. In some studies this may be related to chance, as imbalanced numbers of specimens from each serotype were included, leading to wider confidence intervals for sensitivity estimates in the smaller serotype groups. Validation and verification of NS1 antigen detection assays should include specimens representative of all serotypes and both primary and secondary infections. This should be with serotypes common in the local context to ensure confidence in the performance of these assays.

An NS1 serotype-specific antigen capture ELISA has also been evaluated for rapid serotyping [64,69, 85]. An ELISA with polyclonal NS1 antigen and serotype-specific detector antibodies had comparable diagnostic and analytic sensitivity and specificity to a commercial NS1 Ag ELISA and no cross-reactivity with other flaviviruses *in vitro*, though field evaluation did not occur in a setting with other circulating flaviviruses [64]. A subsequent study in specimens from South America, Central America and India used serotype specific capture and detection antibodies, which were region-specific, and had similar sensitivity with variable specificity (68 –94 % depending on serotype) [85]. Development of these assays would provide additional information in settings where molecular methods for serotyping are not available or not affordable, and has the advantage of speed over classical serotyping based on virus neutralization assays. After a period of time and with a reliable laboratory information management system, they may assist with determining whether a patient has primary or secondary DENV infection, if testing reveals a different serotype from that noted at a previous infection. The disadvantage of this approach is the inability to obtain RNA for sequencing for more in-depth epidemiologic study, though targeted specimens of new serotypes could be referred for further analysis.

NS1 antigen detection by Ag capture ELISA and ICT is primarily used on serum or plasma. Dried blood-spot specimens of both venous and capillary blood also have good sensitivity for ELISA [84,86, 87]. NS1 Ag capture ELISA can also be used on urine and oral fluid, with a notable decrease in sensitivity. This had a diagnostic sensitivity of up to 42 % in saliva and 24 % in urine on the fourth day post-onset of febrile illness. This compared with >80 % for plasma and was lower than RT-PCR on the same specimens. There was also a short window of antigenuria (median 1 day) [38]. Another study reported sensitivity of 64.7 % for saliva in a commercial NS1 antigen capture assay, using oral swabs rather than via direct salivation [87]. It used a comparator population with confirmed NS1 antigenemia in blood, whereas the other study used a broader diagnostic criterion where any combination of positive tests confirmed a diagnosis of dengue [38]. Comparing results with a population confirmed to have NS1 antigenemia may have resulted in a higher estimate. The utility of saliva and urine as a first choice specimen for NS1 Ag ELISA are limited, but a positive result may allow a diagnosis if no other specimen and test combination is available.

Monoclonal antibody 4H2 binds to NS1 antigen and can be used for immunohistochemistry to demonstrate DENV-infected cells in tissue [88]. The sensitivity of 77 % for liver and spleen tissue compared favourably with mouse polyclonal DENV antibodies (sensitivity 51 %) and no cross-reactivity was seen with other flaviviruses or patients with other inflammatory conditions (though sample sizes were small). However, another study found higher sensitivity of RT-PCR (49 %) than immunohistochemistry (40 %) [89].

Overall, owing to high specificity and context-dependent sensitivity, NS1 antigen is a useful test to confirm a diagnosis of DENV infection but cannot be used to exclude infection.

## Molecular methods

Antigen detection via nucleic acid amplification testing (NAAT) is faster than viral culture, and typing can be performed more quickly than by serologic methods. In addition, nucleic acid product may be sequenced for more granular epidemiologic investigation, which may lead to public health benefits.

### Specimens for molecular diagnosis

As an RNA virus, DENV is labile and sensitive to deterioration, which is advantageous in reducing the risk of contamination in laboratory assays, but can reduce the sensitivity of molecular assays if pre-analytical handling is inappropriate [16]. A 37% reduction in specimens testing RT-PCR positive after 48 h storage refrigerated at 2–8 °C was observed, and and 20 % reduction after 15 days storage at −30 °C [39]. Whichever specimen is chosen, appropriate handling is required to optimize yield.

Plasma or serum are most commonly used for NAAT, though detection may be possible for several weeks longer in whole blood [90]. A nasopharyngeal swab was a poorly sensitive specimen (68 % compared with PCR of blood) in a small study, but may be suitable for situations when phlebotomy is not possible [91]. Dried serum [92], venous [87] or capillary [86] blood spot may also be used for diagnostic purposes [87] and epidemiologic studies [12] but are also less sensitive. However, they have advantages in easier and cheaper collection, transport and storage that is not dependent on an intact cold chain. Capillary blood can be collected from a finger prick, and so may be more acceptable in paediatric patients. Dried serum blot with RNA stabilizer had equivalent detection to venous serum (100 % on 20 samples) and can be stored and transported at room temperature [93], but requires phlebotomy and more involved sample preparation at collection, so may suit applications where transport and storage cold chains are the primary concern. Given the variety of potential collection types, collection of a blood specimen in some form for molecular diagnostics could be considered in nearly any situation.

Other specimens are occasionally used. Urine was less sensitive than PCR of plasma in the first 5 days of illness (sensitivity <50 % vs >90 %), but had improved sensitivity, approaching parity in later infection with maximum sensitivity of 66 % at 9–10 days post-onset of fever, and with a longer window of detection up to 13 days in low numbers of patients [38,94]. Saliva, however, had sensitivity around 60 % in the first 4 days of illness and then declined [38]. The prolonged excretion in urine compared with detection in blood may be useful for retrieval of viral material for epidemiologic and public health studies if blood specimens were not obtained early in the illness. Data relating to prolonged excretion are potentially not generalizable to endemic settings where viremia is shorter, particularly in the setting of reinfection. PCR is used less frequently on CSF than serologic tests (though it is unclear whether this relates to access or better diagnostic sensitivity) [37]. Mosquitos and both fresh and formalin-fixed paraffin-embedded human tissues are also described [89,95, 96]. The diagnostic value of alternative specimen types is limited to situations where blood is not available or is negative, or where focal organ infection needs to be confirmed.

### PCR

NAATs such as RT-PCR can be used to detect DENV RNA. Kinetics of viral RNA in primary dengue infection are best exemplified by responses in travellers from non-flavivirus-endemic areas who become infected, as there is presumptively minimal interference from prior antibody, including cross-reactivity from other flaviviruses.

A study of Finnish travellers reported detectable viral RNA in plasma by RT-PCR for a mean 9 days of illness (95 % CI 8–10 days) though 35 % were no longer detectable at day 7 [68]. This differed from studies of primary infection in children [73] and adults [34] in Vietnam, a DENV-endemic area, in which time to resolution of viremia by RT-PCR of plasma was 6 or 7 (IQR 5–8) days. This may relate to the impact of circulating antibody from other flaviviruses or differences in the models used in the Finnish study, as daily viral loads were not performed. In secondary dengue infection, duration of PCR-detected viremia is shorter (5 days in both children and adults) [34]. Presence of IgM reduced viral load and success of viral recovery in culture [35,97], which may explain why the timing of resolution of viremia is earlier in secondary infection.

A small number of asymptomatic household contacts of acute dengue cases had viremia detectable by RT-PCR, with lower viral loads when assessed on dried blood spot. Duration of viremia was found to be up to 2 weeks after a symptomatic index case, but this was highly variable [12]. Duration of PCR positivity may be impacted by age, prior exposure, timing of infection relative to the index case and recency of prior infection. It may also reflect that virus remains detectable longer in whole blood than in plasma [90]. Another study found prolonged detection in capillary dried blood compared with venous phlebotomy specimens [86]. As the venous specimen PCR was performed on serum, this result may also represent increased duration of detection in whole blood rather than prolonged detection in capillary samples over venous.

### PCR methods

Nested RT-PCR for serotyping with dot-blot hybridization was the original molecular method of serotyping [98]. This was subsequently modified to a single tube assay [99], and to a real-time method, with associated improvement in speed, sensitivity and specificity [100,104]. Now commercial assays for DENV and multiplexed methods for joint detection of DENV and other co-circulating arboviruses such as Chikungunya and Zika are available, with excellent diagnostic sensitivity and specificity, and low limits of detection [105,106].

In-house RT-PCRs frequently use well-conserved regions of the 3′ or 5′ untranslated regions (UTR) as targets. Other regions including C-prM, E gene, capsid, NS5 and combinations of these with 3′ and 5′ UTR are used for serotyping [105]. Minimum analytical specificity is<=100 copies/reaction and diagnostic sensitivity and specificity compared with composite clinical reference standards are as high as 100 and 100 %, though varied between 85–100 % sensitive depending on comparator assay and clinical stage of illness [100,102, 104,113]. Many described assays use TaqMan hydrolysis probes, one variant of which has been shown to be less sensitive for DENV4, though a remedial modification is available [114]. Although published verification and validation assays report good sensitivity and specificity, some external quality assurance programmes have detected lack of sensitivity for particular serotypes as well as laboratory performance issues [115]. This issue shows regional variability [116]. Most assays target highly conserved regions but some use genes (such as E gene) that have increased variability [117]. Given this, surveillance using whole-genome sequencing for viral genomic drift will be important to ensure primers and probes remain sensitive. Well-designed assays validated and performed in accordance with a quality-management system generally have high specificity, though false positives may occur from contamination or methodologic issues. RT-qPCR is used as a gold standard comparator in many studies of diagnostic accuracy and owing to its high specificity and good sensitivity can be used to confirm diagnosis of DENV infection, though given the context-dependent sensitivity, cannot be used to exclude infection without further testing.

Multiplex automated PCRs (eg Biofire FilmArray) in combination with other pathogens of relevance for undifferentiated fever with tropical exposure are available, but have poorer sensitivity (80 %) compared with targeted NAAT [16]. Hydrolysis-probe based reverse-transcription-insulated isothermal PCR is also commercially available and can be performed on a handheld portable instrument. In a validation study on patient specimens, it performed favourably compared with NS1 antigen, though was not as sensitive (87 %) as directed PCR [118]

### Isothermal methods

Isothermal nucleic acid amplification methods such as loop-mediated isothermal amplification (LAMP), transcription mediated amplification (TMA) and recombinase polymerase amplification (RPA) have been a focus of research in recent years due to their perceived potential for deployment to lower resource settings. Isothermal methods have the advantages of not requiring thermal cyclers, single tube assays, direct visibility of reaction results and faster assay time when compared with PCR, making their use potentially viable even in isolated settings without reliable power.

Transcription mediated amplification is a sensitive isothermal method that is suitable for high-throughput testing in the laboratory setting, such as in blood transfusion screening [119]. It has been used to screen blood donors during an outbreak in Brazil [15] and may be useful for assessing seroprevalence and seroconversion rates for public health monitoring purposes. A study using TMA to detect virus estimated an 8 day viraemic window, but this was based on estimated background prevalence and seroconversion rates rather than serial measurement, and may be an overestimate rather than a reflection of improved sensitivity of TMA as a methodology [15].

Reverse-transcriptase (RT) LAMP has been considered a good candidate for deployment in point-of-care assays, and several adaptations for portable device have been described [120,123]. Some LAMP assays may be suitable for direct amplification without prior nucleic acid extraction [120,121]. LAMP can be used to differentiate serotypes [124,125] or as a single tube reaction for clinical diagnosis [120,126]. It shows comparable sensitivity and specificity to RT-PCR in clinical samples and has a low limit of detection [126,127], though as for RT-PCR and NS1 antigen detection, these parameters vary by serotype [125]. Multiplex assays with other arboviruses are possible with appropriate detection techniques [120]. However, LAMP (and other isothermal methods) may be prone to spurious amplicon production that may affect analytical specificity [128]. Although LAMP has been considered a good candidate for deployment in POC assays, no studies to date have evaluated a LAMP device under field conditions or on a large scale with patient samples [128], and information provided in some publications is insufficient to evaluate claims made regarding diagnostic validity.

Recombinase polymerase amplification is a rapid isothermal amplification method that uses a probe-based detection method [129]. It is faster than LAMP (<30 min) and runs at a lower temperature, which may increase its portability for field use. Reverse-transcriptase modifications have been described for the detection of DENV using exonuclease probes [130,131]. These studies showed a limit of detection of 11–241 DENV genome copies depending on serotype and assay. There was no evidence of amplification of other flaviviruses and equivalent in-laboratory sensitivity and specificity on both cultured virus and diagnostic specimens to RT-LAMP. There was however both lower sensitivity and specificity than RT-PCR (sensitivity 72–77 %, specificity 97.9–100 %). One assay with modifications for use in low-resource conditions underwent field trials on both laboratory virus and stored patient specimens, with a reduction in performance compared with laboratory use [131]. These studies still required field extraction of RNA rather than direct use on patient specimens. This indicates the difficulty with field application of less complex molecular assays.

A further study assessed the potential of use of RT-RPA in combination with a lateral flow assay and nfo-nuclease probe, without the use of RNA extraction kits [132]. Virus-spiked specimens of human blood, serum and plasma, and archived patient serum specimens were processed with a one-step proprietary reagent before RT-RPA. This assessment indicated poor analytical sensitivity on whole blood compared with serum and plasma. In whole blood, 199 700 copies/ul was the limit of detection. However, 367 copies/ul were detected in for serum and and plasma (similar to that previously described [130,131]). Diagnostic sensitivity (as compared with RT-PCR) on patient serum specimens was low. Sensitivity was 37 % for DENV-1 (though DENV-2 was 100 %), much poorer than the previously reported assays. As overall numbers tested were small, confidence intervals were wide. The assay targeted a conserved region within the NS5 gene [107], whereas the previously reported assays used conserved regions of the 3′UTR [130,131], which may explain the difference in sensitivity.

Although progress has been made in development of isothermal methods for field use, their performance still needs improvement. As with NS1 antigen detection and non-isothermal molecular methods such as PCR, it does not allow exclusion of DENV with a negative result, even in early infection. There are concerns regarding reduced specificity for some assay types, and field application may increase the risk of false positives due to contamination between specimens. Given the relative cost and comparative complexity of current isothermal methods (the best performing methods of which still require RNA extraction), and their limited benefit after 7 days of illness (few specimens were positive by any molecular assay after this time), their cost-effectiveness in the field is still questionable. It may be better in resource-limited contexts to consider referring stable specimens (e.g. serum blots [93]) for molecular analysis to obtain organism confirmation, serotype and genotype information for public health, and to use existing NS1/IgM/IgG combination rapid diagnostic tests (RDTs), which have comparable sensitivity and specificity [133], for acute patient management. Nonetheless, the speed, low power usage and absence of cold chain requirement, and potential for simpler sample processing without RNA extraction, all indicate the future potential of these assays with ongoing refinement.

### Sequencing

Sequencing is an additional tool used to improve understanding of the epidemiology of DENV. Genotypes have been defined by Sanger sequencing of the E gene [134]. Multiple genotypes are recognized within a serotype [135]. With the advent of next generation sequencing (NGS) high-throughput methodologies, a more detailed understanding of viral adaptation and mutation through sequence variation, as well as phylogeny relating to local epidemiology can be appreciated through whole-genome comparison [136]. As DENV are RNA viruses they would be expected to have comparatively high mutation rates due to a lack of proofreading of RNA-dependent RNA polymerase. In the case of DENV this is paradoxically slower, possibly due to evolutionary trade-offs, but useful phylogenetic information can still be obtained [136].

Both long- and short-read NGS methods are in use. Initial approaches to short-read NGS for DENV required purification and comprehensive enrichment [137]. Short-read methods appear to have good sensitivity. Hybridization-based target enrichment was able to detect whole DENV1 and 2 genomes from spiked pooled samples at lower concentrations than would have been expected to be detected by PCR as part of a metagenomics multi-pathogen panel [138]. Further work indicated targeted enrichment using baits could effectively detect whole DENV genomes from patient samples including co-infections with other flaviviruses such as Zika virus [49]. Spiked primer enrichment prior to metagenomic NGS yielded comparable sensitivity to PCR for detection of DENV from patient plasma using long-read methods when an arboviral enrichment panel was used, while retaining sensitivity for non-targeted viruses [139]. Long-read methods have the advantage of speed and are suitable for in-field use [140], though error rates and specificity can be problematic with this methodology. In situations where NGS is not locally available, sequencing can be performed successfully though with significantly reduced sensitivity, from stabilized RNA serum blots. This could improve accessibility in situations where cold chain transport is not easily obtained [93]. Methods continue to be refined as their applicability to different situations are assessed.

Monitoring genotypes of circulating viruses by sequencing is important for multiple reasons. Different viral variants may vary in pathogenicity, although at present separating viral variants from the spread of wild-type viruses is difficult [136]. Additionally, viral mutation may have implications for specificity of NAAT test targets. These may be of relevance for epidemiologic investigations and public health interventions, including immunization [135]. Sequencing could also be used to confirm a diagnosis if false positives in NAAT were suspected. Therefore, NGS (while not currently a primary diagnostic modality) can provide useful early warning of genetic drift to allow for update of test targets, and may in future provide genotyping information with clinical importance. Given improving sensitivity of detection and ability to detect multiple arboviral and other pathogens concurrently as part of a metagenomics approach, while also providing sequence data for epidemiology and clinical assessment, NGS approaches have significant future potential for diagnosis of acute DENV infection and monitoring in outbreaks.

## Antibody detection

Antibody detection is the commonest diagnostic modality requested to support the clinical diagnosis of DENV infection. It is generally performed on serum or plasma, though dried blood spots of venous or capillary blood, saliva and CSF can be used [37,84, 86, 87, 141,143]. Use in diagnosis is hampered by the degree of cross-reactivity between flaviviruses of the viral E protein in serologic assays, against which most IgG and IgM antibodies used in diagnosis are directed [67]. Antibodies against NS1 may show less cross-reactivity, though studies are limited and reach opposing conclusions [144,146]. Cross-reactive E and NS1 antibody would also influence cross-reactivity in total antibody assays. 54 % of patients with virologically confirmed Zika virus infection, confirmed to be previously seronegative to DENV, had antibodies to DENV IgG, presumably due to crossreactivity [147]. Comparison with neutralization methods suggests variable sensitivity of serology by DENV serotype, as previously discussed for NS1 antigen detection [148]. In addition to this, many serologic assays do not distinguish viral serotype, meaning prior DENV infection may also affect which antibodies are detectable and when. Unless multiplexed methods are used or confirmed by other more specific assays, diagnosis by serology should screen for other flaviviruses to which the patient may have been exposed concurrently where possible [149].

### Microsphere Immunoassay

Microsphere Immunoassay (MIA) has been developed as a solution to the problem of significant cross-reactivity between flavivirus viral E proteins IgM and IgG. As a platform compatible with multiplex analysis, it can be used to screen and differentiate IgM and IgG for multiple arboviruses simultaneously. Differentiation is performed using statistical methods while analysing a panel of arboviruses, as cross-reactivity is still seen within the assay. Simultaneous testing and internal controls allow analysis for determination of the correct infecting organism [141]. These panels can be broad, regional panels (for use in febrile travellers in non-endemic settings), or focal panels of common local organisms. In addition, high-throughput screening is possible, though specialized equipment is required. These assays are not currently commercially available.

Initial performance for DENV using recombinant serotype 2 and 3 antigens achieved correct classification for 98 % of IgM result and 99 % of IgG results in a logitboost model as part of a regional antigen panel [141]. When considering DENV without other antigens, 94 % of IgM specimens and 84 % of IgG specimens would have been correctly classified by cut-offs developed by a receiver operating characteristic curve. While other viruses had detectable reactions to the DENV antigen, the mean fluorescence intensity was >2.5 times higher in truly DENV positive serum, allowing differentiation. Similar findings were found for other flaviviruses.

Antibodies against NS1 IgG show similar cross-reactivity with other flaviviruses as E protein antibodies, causing similar problems with using them for serodiagnosis [144]. While a recombinant NS1 antigen ELISA did not show cross-reactivity with Zika virus, other arboviruses were not assessed [150]. However, a multiplex microsphere immunoassay against NS1 IgG was able to distinguish DENV from Zika and West Nile Virus (WNV), including in the context of secondary infection, with sensitivity of 94.5 % and specificity of 97.2 % (though serotype specificity was lower) [151]. Clearly, caution is required for serodiagnosis if more than one arbovirus is potentially present in a patient’s specimens or exposure history, but multiplex MIA shows promise for improved differentiation, and NS1 antibodies merit further investigation.

### EIA for IgM

IgM antibody in serum generally becomes positive by day 6–8 of illness in primary DENV infection but may be detected as early as 3 days in some patients. It peaks at 2 weeks, and is detectable for up to 3 months [152,154]. IgM tires are lower in secondary infection, decline sooner and may not be detectable at all in some patients, depending on the timing of sample collection [155]. Titres may also be reduced if the patient has previously been infected with another flavivirus [11].

IgM antibody Capture ELISAs (MAC-ELISA) are the assays in most widespread use as they have improved sensitivity and specificity compared with other EIA methods. A schematic is given in Fig. 2b. This is due to targeted binding of IgM, which reduces nonspecific binding and reduces competition from IgG [156]. Performance of assays in quality assurance programme testing of IgM ELISA showed 88.9 % accuracy, confirming the sensitivity, specificity and comparative ease of use of the method [116]

Commercial IgM ELISA kits were evaluated on reference sera representing the four serotypes [157]. The best performing kits had sensitivities of 99 % and specificities of 79.9–86.6 %, with cross-reactivity seen with malaria, leptospirosis, and rheumatoid factor, though other flaviviruses were not assessed. However, cross-reactivity with Japanese Encephalitis virus (JEV) and other flaviviruses may be higher. Sixty two percent of acute PRNT-confirmed JEV-infected patients had positive or equivocal DENV IgM, though the ELISA index was lower than for JEV IgM [158]. In another study, 34 % of Zika virus confirmed specimens were positive in a DENV MAC-ELISA [159]. This reinforces the need to screen for multiple flaviviruses concurrently in the acute setting if more specific methods (such as DENV NS1 antigen or NAAT) are not in use.

A Duo MAC-ELISA using concurrent testing of Zika and DENV with an optical density (OD) ratio based cut-off showed excellent discrimination of DENV and Zika from day 8 post-onset of illness, equivalent sensitivity to commercial DENV MAC-ELISA and minimal cross-reactivity with other flaviviruses [159]. This approach could be useful where multiplex MIA is not available, but would need to be validated in the location of use to assess appropriate OD ratio cut-offs and to assess specificity.

### EIA for IgG

IgG of E protein may be detectable by ELISA as early as day 12–15 in primary infection [152,153]. In secondary infection it is detectable at day 4 in some and day 7 in most patients [153], Several indirect ELISA and IgG capture ELISA assays are commercially available [11,116, 160]. Most are validated for serum, some for plasma, and performance of a commonly used non-commercial protocol in plasma was equivalent to serum [161]. Performance of commercially available and in-house EIAs in quality assurance programme testing showed 100 % accuracy, confirming their sensitivity, specificity and ease of use [116].

The monoclonal antibody-based IgG Capture ELISA has improved specificity compared with the indirect ELISA due to less capture of non-specific background IgG, though cross-reactive IgG will also be captured [162]. In secondary DENV infection, anamnestic IgG is the primary antibody response, and is directed at the previously infecting serotype. However, due to cross-reactivity between flavivirus E proteins, a similar pattern may be seen if a patient has previously had another flavivirus infection rather than prior DENV infection. In total, 87.5 % of patients with acute PRNT-confirmed JEV had positive or equivocal DENV IgG, and 50 % were also ELISA positive for another flavivirus [158]. Another study noted cross-reactivity with Chikungunya virus positive specimens ^160^ IgG Capture ELISA using 2H2 monoclonal antibody compared favourably with PRNT when assessing for evidence of prior exposure to DENV for assessing suitability for DENV vaccination [163]. Sensitivity and specificity of the IgG Capture ELISA (compared with PRNT) was 91 and 91 %. Performance of an indirect IgG ELISA (compared with FRNT) was similar, with sensitivity 95 % and specificity 93 % [148]. PRNT and FRNT comparison suggested that JEV and Zika virus cross-reactivity was the reason for less than perfect specificity, again reinforcing the difficulty with serodiagnosis where other flaviviruses circulate.

Development of more specific assays for other flavivirus, such as NS1 blockade of binding ELISAs may also help distinguish them from acute DENV infections when specificity is a concern [164]. ELISAs using virus-like particles (VLP) in which cross-reactive epitopes from the E protein have been removed also showed significantly less cross-reactivity for both IgM and IgG for Zika, DENV1 and WNV than direct ELISA using wild-type Zika or DENV VLP [165]. Comparison of ratios of optical density of wild-type VLP and modified E protein VLP for Zika, DENV1 and WNV assisted with distinguishing primary and secondary DENV1, primary Zika and Zika virus with prior DENV infection. If this approach were further developed to consider DENV serotype and validated on a wider range of patient specimens, it could allow a focussed high-throughput method of distinguishing multiple arboviruses without the specialized equipment required for MIA.

A NS1-antibody IgG competitive ELISA using serotype-specific aptamers incorporating non-natural bases has been described as a method to assess serotype of infection [166]. In primary infection, anti-NS1 IgG specific to the infecting serotype could be detected after a minimum of 1 week post-onset of fever. In secondary infection it was detectable as early as 3 days post-onset of fever, though reacted to the previously infecting serotype. However, this assay was only tested on specimens from 11 patients, discrepancies in agreement with existing DENV IgG assays (both EIA and ICT) were seen. Very limited assessment against well-characterized sera for other flaviviruses was undertaken. Further evaluation in well-characterized sera and patient specimens is required to confirm specificity of this assay.

### Inhibition ELISA

Inhibition ELISA (IE) has the advantage over IgG capture ELISA of yielding an inhibition titre (or mean percentage inhibition if desired). This allows a degree of functional assessment of antibody binding, which may be of importance for assessment of immune competence, rather than just history of exposure. It has similar performance to haemagglutination inhibition (HI) in sensitivity and specificity [167,168]. While IE does not provide an assessment of neutralisation capacity, IE titres correlated to percentage PRNT titres and to mean 50 % neutralizing titre (NT_50_) in a flow-cytometry-based neutralization assay [14]. IE is less technically complex to perform than HI and suitable for high-throughput testing, favouring its use in screening (such as pre-vaccination).

Monoclonal antibody studies indicated that IE primarily measures inhibition from antibodies such as 4G2 (fusion loop) and 2H2 (prM) that are involved in cross-reacting neutralizing responses implicated in ADE. Titre by IE (against balanced mixed DENV antigens) has been used to stratify risk for ADE in secondary DENV infection [14]. Intermediate titres by IE indicated higher risk of ADE. Use of this test may allow further stratification of benefit from vaccination.

### Haemagglutination inhibition

Haemagglutination inhibition has been used for the serologic diagnosis of DENV infection since prior to availability of ELISA, and can be used to differentiate primary and secondary infection based on inhibition titre [11]. It cannot be used to differentiate infecting serotype [169]. However, while sensitive, it suffers from similar specificity issues to other serologic assays due to broad cross-reactivity of flavivirus IgG [170]. The ratio of IgM/IgG in capture ELISA, and presence of acute IgG were both found to better discriminate primary and secondary DENV than HI [171,172]. Given the comparative ease of use of ELISA methods and their commercial availability, this assay is used comparatively less frequently [116].

### Other antibodies

IgE has been explored as a diagnostic target because of its potentially increased sensitivity in the first 3 days of infection when IgM may not yet be detectable. An IgE capture ELISA achieved a sensitivity of 82 % and specificity of 77 % compared with RT-PCR in a mixed cohort of DENV1 and DENV2 confirmed infection [173]. No analysis of efficacy in secondary vs primary infection was undertaken. NS1 antigen was not compared in this study, but based on prior work it appears that sensitivity would be at least equivalent if not better, and specificity significantly better [66,70, 78]. Unless a clear role for IgE in DENV pathogenesis is found giving clinical utility to its measurement, NS1 antigen appears to be a more useful diagnostic test in early infection.

IgA has also been a target of study for diagnosis in early infection as its shorter persistence than IgM may be useful in diagnosing reinfection in countries where multiple serotypes circulate or confirming timing of infection for epidemiologic analysis [174]. A meta-analysis of studies investigating IgA capture ELISA (including one commercial assay), IgA antigen capture ELISA, IgA immunofluorescence assay and IgA ICTs for acute diagnosis of primary and secondary dengue using serum compared with various reference standards found significant heterogeneity [175]. Pooled sensitivity was 74 %, and pooled specificity was 95 % and was not significantly different between ICTs and ELISAs. However, pooled sensitivity was 86 % in the subset of patients in which specimens were collected on day 4 or later of illness, and pooled sensitivity 92 %. Studies were undertaken before the widespread circulation of Zika virus, which could impact specificity. Additionally, the effect of serotype was not considered. A study of IgA antigen capture ELISA on dried blood spot detected it in 73 % of convalescent specimens in patients with secondary DENV [84]. There was no difference between serotypes however only one serotype 4 infection was included. Performance was better for both IgM and NS1 antigen with both detected in 92 % of early convalescent specimens. Given difficulties with diagnosis of secondary DENV, further assessment of IgA serum assays for specificity in secondary DENV in the light of other circulating flaviviruses would be of interest.

### Primary vs secondary dengue – interpretation of serology

As secondary DENV infection with a different serotype increases the risk of severe dengue, differentiating a primary and secondary infection is clinically important. Ideally, serotype confirmation by virus isolation or RT-PCR would be performed for each infection, but this is frequently unavailable or impractical. However, serologic criteria can be used to assess whether an infection is likely to be primary or secondary.

Differentiating primary and secondary DENV infection is possible by assessing the ratio of IgM to IgG in a single acute specimen. The accuracy of ratios likely decreases after 30 days post-infection and alternative approaches should be used for less acute specimens [176]. In acute samples single IgM/IgG ratio of>=1.2 was consistent with primary DENV infection and <1.2 with secondary DENV infection, with >90 % agreement with the prior method in use (HI) in one study [171] and with a composite serologic standard in another [177]. Other studies have found different cut-offs, varying between 1.20 and 2 [176,178]. A study using PRNT predominant serotype at 6 months as compared with NAAT-confirmed serotype at time of infection to define primary and secondary DENV infection found that the optimal cut-off declined with time after infection but was broadly consistent with the cut-offs previously quoted [179]. As cut-offs for ratios are not standardized between assays, they require in-house validation [11].

HI titres of greater than or equal to 1 : 2560 were also previously used to diagnose secondary DENV, but required two specimens tested in parallel, and were confounded by other flavivirus infections due to cross-reactivity [171].

IgG avidity using chaotropic dissociation with either indirect or capture ELISA can be used to differentiate primary and secondary dengue within 30 days of illness and may classify patients better than IgM/IgG ratio after 30 days [176,177, 180]. Agreement with IgM/IgG ratio is good in acute specimens, but none of the studies assessing avidity used patients with virologically confirmed infection. Kinetics of IgG avidity in secondary DENV2 infection are reported to be similar to DENV3 infection [181], but assessment of sensitivity of avidity for diagnosis of secondary infection in different serotypes has not been performed. Further assessment is also needed as to the specificity of this approach in patients with primary DENV and a history of prior flavivirus infection, in the setting where multiple flaviviruses circulate.

Biolayer interferometry avidity assays have been used to assess vaccine candidates using DENV 1–4 VLP [182]. Given an association between lower antibody avidity and severity of DENV infection [181], and evidence of intermediate titres by inhibition increasing the risk of ADE [14], it is possible that avidity assays might be useful in future for assessing the risk of developing severe DENV disease and therefore whether vaccination or revaccination is indicated.

Selection of an appropriate serologic test algorithm to confirm a diagnosis of DENV and classify a patient’s illness as primary or secondary infection requires understanding of the detectable biomarkers as time passes after infection and is discussed further later in this review. In addition, knowledge of the background incidence and prevalence of DENV and other flaviviruses is essential. It may be best determined with a combination of tests on a single specimen, though in acute illness in low-prevalence settings a single test may have good diagnostic performance [83,179, 183, 184].

### Serology in the setting of planned immunisation

WHO recommends screening for presence of DENV IgG prior to immunization with live recombinant vaccines known to increase the risk of ADE [29]. Higher test specificity is required in lower-prevalence environments. In environments with high-transmission settings, rapid diagnostic tests designed for acute use might be acceptable despite a lower sensitivity and specificity [29]. Alternatively, population-based surveillance could be used to confirm appropriateness of vaccine use [27]. An assessment of indirect IgG ELISA compared with FRNT found that it was least sensitive (77 %) in those with FRNT suggestive of previous single serotype infection, who would be most expected to benefit from vaccination [148]. Specificity was 93 %, due to cross-reactivity with Zika virus and JEV, which does not meet WHO-specified target specificity of 98 %, however. NS1 antibody may be useful in discriminating DENV-naïve patients to exclude them from vaccination, and showed minimal interference from other flaviviruses, though specimens predate the Zika virus outbreak [185]. Further validation in a cohort of sera with broader flavivirus exposure would be beneficial.

## Differentiating infection from vaccination

Serology may become less informative for acute diagnosis as vaccination is implemented as a commercially available DENV vaccine expresses the DENV E protein. Most current antibody tests measure antibodies against this epitope. Data from trials of a modified live recombinant tetravalent DENV vaccine found that there was increased prevalence of IgM and IgG seropositivity in febrile vaccinated patients without virologically confirmed DENV compared with controls. The IgM and IgG results would have led to diagnosis of DENV if serologic criteria were used, but in the absence of virologic confirmation was considered likely vaccine-induced antibody and therefore falsely positive. Vaccination therefore reduced the specificity of IgM and IgG criteria as diagnostic markers for DENV infection, likely due to the presence of vaccine-induced antibody. This was more pronounced in patients who were seronegative prior to vaccination [186,187]. Antibody to DENV NS1, which is not expressed by the vaccine, has been evaluated in pre- and post-vaccination specimens [185]. Vaccination did not cause seroconversion, but NS1 antibodies were detectable in patients with a history of prior infection. This assay would be helpful to detect a first infection in vaccinated patients, but as at present vaccines are only for use in those with a history of confirmed DENV infection they are unlikely to be useful in the current clinical scenario. Additionally, some authors raise concerns that NS1 antibody is not specific for DENV [144,145]. NS1-antibody competititive ELISAs have not had specificity widely evaluated in patient specimens or against other flaviviruses [166]. ELISA for IgM and IgG antibody to E proteins as a single diagnostic marker may not be reliable in a vaccinated population. Confirmation of infection with NS1 antigen or NAAT, or paired IgM and IgG serology using a quantitative method, will be important for confirming a DENV diagnosis in the era of vaccination. This is similar in some ways to the post-vaccination diagnosis of pertussis, Q fever and varicella zoster infections, where serology alone may not easily distinguish between vaccine response and infection.

## Rapid diagnostic tests

Rapid diagnostic tests are used for diagnosis of acute Dengue at the point of patient care, or in smaller laboratories which do not have the ability to perform EIAs and NAAT [188]. The use of IgG assays has also been considered as part of pre-vaccination screening for evidence of prior exposure [188].

Most RDT in current use are ICTs in cartridge format, either for Dengue IgM and IgG, NS1 antigen, or a combination of these. A recent systematic review and meta-analysis assessed the performance of IgA, IgM/IgG, NS1 antigen, and combination NS1/IgM/IgG assays in 40 studies published prior to October 2019 [133]. Considerable heterogeneity was observed in sensitivity estimates between the pooled studies, especially for NS1 antigen potentially relating to both operator factors and variability in populations to which the test was applied and variability in the gold standard referenced. The overall performance was best for the NS1/IgM/IgG tests, which achieved a pooled sensitivity estimate of 91 %(95 % CI 84–95) and specificity of 96 %(95 % CI 91–98). This likely reflects their spectrum of activity. NS1 antigen tests would be most accurate in early acute infection (up to 1 week post-onset of symptoms), whereas IgM/IgG combination tests would have better performance in convalescent specimens. As patients may have been at variable stages of illness when tested in the underlying studies, it is unsurprising that a test that will detect patients in both early and later stages of infection has better performance. While IgA assays had a similar pooled sensitivity (88 %, 95 % CI 73–95 %), the specificity was poorer (90 %, 95 % CI 78–96 %). The wider confidence intervals may reflect the smaller numbers assessed for this assay, and is within the 95 % confidence intervals of that previously reported [175]. Overall, the combination NS1 antigen/IgM/IgG rapid diagnostic tests are a useful diagnostic tool for patients presenting with suspected DENV infection, where easy access to EIAs and NAATs is not available.

The performance of IgG RDT assays as compared with a reference method (generally ELISA) for identifying patients with prior DENV infection for the purposes of determining the safety of DENV vaccination was assessed [188]. None of the ten studies reviewed were performed for the purpose of diagnosing previous DENV infection and many of the included specimens were from patients with acute infection, though almost half included some patients with known prior DENV infection. Concerningly, the specificity of RDTs in convalescent samples and patients with confirmed primary DENV infection was as low as 58 % for some assays with wide confidence intervals. In convalescent specimens, the lowest sensitivity reported was 50 %, though most were >80 %. Given the potential for severe DENV infection in those vaccinated without prior antibody, high specificity is of importance in ensuring that vaccines do not cause harm [189]. Wide variability was seen between specificity estimates from studies where the same assay was used (specificity of 100 % in one study and 85 % in another), suggesting potential regional cross-reactivity with other flaviviruses, or issues with methodology and repeatability.

As most of the evidence assessed in the systematic review was indirect and the assays compared were not performed specifically for assessing suitability for vaccination, other studies have prospectively compared IgG RDTs for suitability as a pre-vaccination assessment against a PRNT or ELISA comparator [190,192]. In contrast to the previously reported analysis, these prospective studies indicated excellent specificity (generally >98 % for IgG in all samples) and variable and sometimes poor sensitivity (39–69 %). The studies performed under laboratory conditions appeared to be more sensitive than those performed in the field. There was heterogeneity of performance of the same assay in different studies. Those assessing specificity in the setting of other flavivirus exposure or vaccination found little evidence of cross-reactivity [193]. This also contrasts with the previous review and suggests methodology issues or perhaps reflects different prevalences of other flaviviruses at the time of specimen collection. Performance appeared better on serum than venous blood, and with longer assay run time, but at the expense of specificity which was reduced to 94 %. Sensitivity issues may relate to the design of these assays for detection of acute infection, with higher limits of detection [194]. Prior to using IgG acute diagnostic assays for pre-vaccination screening, local validation should be performed to ensure adequate sensitivity and specificity, as performance may relate to IgG threshold of detection for POC tests and therefore be better in regions where DENV has recently been circulating. However, dedicated assays perform better and should be used where possible.

It is unlikely that the IgG RDT designed for use in acute infection can be safely used to determine suitability for vaccination. However, this will depend on field evaluation of sensitivity and specificity parameters and the prevalence in the population, as increasing prevalence will lower the negative predictive value of a test with poor specificity. This means more patients could potentially be vaccinated when not immune, potentially resulting in severe dengue if later exposed. It is therefore not an ideal candidate in determining seropositivity to DENV. The paradox, however, is that at a population level, higher benefit of vaccination is likely to accrue in high prevalence regions where the risk of reinfection is higher and a poor sensitivity test with high specificity would incorrectly exclude many from vaccination [195]. In addition, use in lower-prevalence populations would have a low positive predictive value (even with sensitivity of >80 %), leading to equally poor confidence in providing immunization. These non-dedicated RDT should only be used with caution in assessing suitability for vaccination based on current test performance.

Fluorescent automated antibody detection POC RDTs IgG have been assessed relative to FRNT for pre-vaccination screening in a high-prevalence population [196], although this was done in the laboratory and not the field. Sensitivity of 91 % and specificity of 90 % were achieved (less than the target sensitivity and specificity of 98 and 95 %, respectively), though confidence intervals were wide owing to widespread seropositivity. Sensitivity was worst (51 %) in patients with evidence of only one prior DENV infection, which is the patient group expected to benefit most from vaccination.

An IgG RDT designed for use in pre-vaccination screening has been developed. It is reported as 95 % sensitive (88 % in prior monotypic infection and 98 % in multitypic infection) and 98 % specific [194]. There was no significant difference in serotype sensitivity for prior monotypic infection [197]. There was minimal cross-reactivity with other flaviviruses. However, studies of this assay used archived serum samples rather than blood and were performed in the laboratory. Prior studies indicate lower sensitivity of RDT from whole blood, and in field use [191]. If the laboratory performance can be replicated under field conditions, and with whole blood, this assay could be a valuable pre-vaccination screening tool.

## Diagnostic algorithms for acute dengue infection

The optimal combination of diagnostic tests for acute DENV infection depends heavily on the context. Patient vaccination history, flavivirus infection history, duration of illness, presence of other flaviviruses in the area of acquisition, and test availability all need to be considered.

An important component of test interpretation is the pre-test probability of infection, and this will be higher in endemic or hyper-endemic areas than in non-endemic areas. The pre-test probability of infection, and any history of prior infection, will assist with choosing an appropriate combination of assays. In low-prevalence contexts, testing may be delayed due to delay in accessing testing until after returning from travel, or due to reduced clinical suspicion associated with not obtaining an appropriate travel history. This will also influence the appropriate diagnostic algorithm.

No individual test (antigen, antibody or molecular) can exclude acute DENV infection at present. Similarly, confirming DENV infection can be challenging. However, combinations of sensitive tests chosen with the patient’s history of symptoms and history of prior flavivirus infection in mind can increase confidence in a negative, or positive, result.

Expert groups have developed diagnostic algorithms to suit various circumstances [47]. These require nuanced interpretation. In a well-resourced setting in early acute infection, NS1 antigen detection, in combination with IgM and IgG is a reasonable starting point provided there is high confidence that DENV is the only flavivirus to which the patient may have been exposed. NAAT for flaviviruses to which the patient was potentially exposed could be considered as an alternative, as these tests have high specificity and sensitivity in early acute infection [61]. This is the WHO’s preferred approach [198]. An alternative would be a screening MIA if available, with NAAT confirmation if desired. NS1 antigen could be substituted for DENV NAAT in many cases [83], though multiplex RT-PCR screening may be more efficient if multiple flaviviruses are to be tested. If initial serology is negative and NAAT does not detect flavivirus RNA, paired IgM and IgG EIA for relevant flaviviruses should be performed. In situations where confirmation of the infecting organism is highly desirable such as potential antenatal Zika virus infection, PRNT could be considered if NAAT were not available. It is acknowledged that in settings or patients with prior flavivirus exposure, specificity will be reduced.

Dengue is endemic in a large number of less well-resourced countries. A pragmatic approach to a DENV diagnostic algorithm is required. In these settings, the tests available and local flavivirus epidemiology will influence the approach. However, in early febrile illness a combination of NS1 antigen detection, IgM and IgG is likely to detect the majority of infections, whether primary or secondary. The use of IgM and IgG alone has limitations but will detect early and first infection in many cases, provided other arboviruses are not circulating. An IgM/IgG ratio may be used to assess the likelihood of primary or secondary DENV infection, as previously discussed. The use of IgM and IgG has the significant advantage of not relying on the relatively short period in which NS1 antigen and DENV RNA are detectable, particularly in secondary infection.

However, when resources allow, use of NS1 antigen or NAAT will be increasingly important in patients with a history of vaccination. NS1, IgM and IgG are available as point of care tests to improve access. RT-LAMP in combination with IgM/IgG ELISA detected 97 % of acute cases, and RT-LAMP is becoming increasingly accessible to less well-resourced environments [126]. A multiplex automated PCR had equivalent sensitivity to an NS1 antigen rapid diagnostic test for diagnosis as a single specimen, but was unsurprisingly less sensitive than the combination of RDT, paired serology and PCR, reinforcing that diagnostic certainty often requires multiple samples and does not always require the most expensive tests [16]. If NAAT are not available locally, consideration of stabilized serum blots for referral to a reference laboratory for RT-PCR or sequencing may be useful for epidemiologic assessment of circulating genotypes.

Later febrile illness in a patient from a non-endemic region may still be successfully diagnosed by NAAT, IgM and IgG in some cases, though sensitivity will be lower. Finnish data reported 91 % of presumed previously flavivirus naïve patients were RNA positive at day 2 of illness, but combination testing with NS1 antigen, IgM and IgG improved the overall sensitivity of detection of infection to over 97 % during the initial 3 weeks of illness [68]. Due to shorter periods of viremia and antigenemia in secondary infections, and possibly in patients with prior exposure to other flaviviruses, this result cannot be generalized to endemic settings.

In endemic settings where later confirmation of the infecting organism is relevant for decisions regarding vaccination or local epidemiologic reporting, IgM/IgG ratios, IgG avidity and indirect IgG ELISA should be considered.

## Conclusion

Significant change and development in diagnostic testing for DENV has occurred in the context of new technologies, other circulating flaviviruses, and the advent of vaccination. The range of assays and specimen types available increases the likelihood that molecular diagnostics will become available to a wide range of sites of patient care. However, the role of serologic assays and NS1 antigen have not yet been supplanted in many cases. Vaccination will impact the applicability of workhorse serologic methods. Testing strategies should be considered in advance in areas where vaccination is to be used.

## Abbreviations

ADE: antibody-dependent enhancement
Ag: antigen
CSF: cerebrospinal fluid
CT: cycle threshold
DENV: Dengue virus
E: envelope
EIA: enzyme immunoassay
ELISA: enzyme-linked immunosorbent assay
FC: flow cytometry
FRNT: focus reduction neutralization test
HI: haemagglutination inhibition
HRP: horseradish peroxidase
ICT: immunochromatographic test
IE: inhibition enzyme-linked immunosorbent assay
IgA: immunoglobulin A
IgE: immunoglobulin E
IgG: immunoglobulin G
IgM: immunoglobulin M
JEV: Japanese Encephalitis virus
LAMP: loop-mediated isothermal amplification
mAb: monoclonal antibody
MAC-ELISA: IgM antibody capture enzyme-linked immunosorbent assay
MIA: microsphere immunoassay
NAAT: nucleic acid amplification test
NGS: next generation sequencing
NS: non-structural protein
NT_50_: 50% mean neutralising titre
OD: optical density
PCR: polymerase chain reaction
POC: point-of-care
PRNT: plaque reduction neutralization test
qPCR: real time polymerase chain reaction
RDT: rapid diagnostic test
RNA: ribonucleic acid
RPA: recombinase polymerase amplification
RT: reverse transcriptase
RT-PCR: reverse transcriptase polymerase chain reaction
RVP: reporter virus particle
TMA: transcription mediated amplification
UTR: untranslated region
VLP: virus like particles
WHO: World Health Organisation
WNV: West Nile virus
